# Monitor, a Vibrotactile Aid for Environmental Perception: A Field Evaluation by Four People with Severe Hearing and Vision Impairment

**DOI:** 10.1155/2013/206734

**Published:** 2013-06-19

**Authors:** Parivash Ranjbar, Ingeborg Stenström

**Affiliations:** ^1^Audiological Research Centre, Örebro University Hospital, 701 85 Örebro, Sweden; ^2^School of Health and Medical Sciences, Örebro University, Fakultetsgatan 1, 70281 Örebro, Sweden; ^3^Campus Alfred Nobel, Örebro University, Karlavägen 16, 69141 Karlskoga, Sweden; ^4^Department of Ophthalmology, Örebro University Hospital, 701 85 Örebro, Sweden

## Abstract

Monitor is a portable vibrotactile aid to improve the ability of people with severe hearing impairment or deafblindness to detect, identify, and recognize the direction of sound-producing events. It transforms and adapts sounds to the frequency sensitivity range of the skin. The aid was evaluated in the field. Four females (44–54 years) with Usher Syndrome I (three with tunnel vision and one with only light perception) tested the aid at home and in traffic in three different field studies: without Monitor, with Monitor with an omnidirectional microphone, and with Monitor with a directional microphone. The tests were video-documented, and the two field studies with Monitor were initiated after five weeks of training. The detection scores with omnidirectional and directional microphones were 100% for three participants and above 57% for one, both in their home and traffic environments. In the home environment the identification scores with the omnidirectional microphone were 70%–97% and 58%–95% with the directional microphone. The corresponding values in traffic were 29%–100% and 65%–100%, respectively. Their direction perception was improved to some extent by both microphones. Monitor improved the ability of people with deafblindness to detect, identify, and recognize the direction of events producing sounds.

## 1. Introduction

Monitor is a device developed to give people with severe hearing impairment (HI) or deafblindness (DB) access to more information about events in their surroundings. The aid, Monitor, uses the vibratory sense and is programmed to handle environmental sounds in contrast to other vibratory aids designed for speech signals [1, 2]. It detects sounds from events picked up by a microphone, adapts the sound to the frequency sensitivity range of the skin using algorithms developed based on modulating, transposition, or filtering principles, and translates the signal as vibrations. The person sensing the vibrations can detect and identify the character and direction of a sound source. 

The previous nonportable version of Monitor was evaluated by people with normal hearing, profound deafness, and with blindfolded deaf people in various laboratory and field studies [3–7]. The results showed that Monitor consistently improved the ability of blindfolded deaf people to detect, identify, and recognize the direction of ongoing events producing sounds at home and in traffic. Four different algorithms, based on modulating and transposing principles, were found to be good candidates to be implemented in a portable vibrotactile aid for persons with DB. In this current study, Monitor's design was made portable by implementing a specific algorithm (one of the four selected in the laboratory) in a cell phone for a specific person with DB. The three microphones in a headband are also reduced to one microphone with two settings, omnidirectional and directional. After a period of training, the portable Monitor will be evaluated in this current study in a realistic environment by people with DB.

There are about 400 people born deafblind in Sweden. About 1600 (<65 years) became deafblind due to more than 30 progressive hereditary diseases affecting vision and hearing. The largest group, about 300 people, have Usher's Syndrome type I (US I). The largest group of DB, estimated at about 30,000–40,000 people, is those over age 65 with a serious combination of vision and hearing impairments [8]. In Sweden and the northern countries, the definition of DB is based on the individual's special needs and not the degrees of vision and hearing impairment. The Nordic definition of DB is as follows.

*“Deafblindness is a Distinct Disability: Deafblindness is a combined vision and hearing disability. It limits activities of a person and restricts full participation in society to such a degree that society is required to facilitate specific services, environmental alterations and/or technology [9].” *

People with DB belong to the category of persons with severe disabilities. The functional difficulties associated with DB are as follows.

Communication and social interaction: the ability to communicate and exchange information with people in their surroundings.

Mobility: the ability to move and orient themselves in their surroundings.

Management of activities of daily living: the ability to independently perform daily activities.

Environmental perception: the ability to perceive ongoing events in the environment and thereby implement appropriate forward planning and control (adapt to and influence).

People with DB do not know what is going on around them. They want to know about ongoing events, such as a fire alarm, and objects below their visual field, for example, children, dog, or baby carriage, so that they do not stumble, or if they are in the path of a vehicle.

Usually they have to use different senses, alone or combined, in different situations. They use information from vibrations, smell, taste, draught (air current), and temperature differences to detect events. Vision and touch are mainly used to identify the events [10].

Hearing aids (HAS) and vibrotactile aids, for example, Tactaid II and Minivib, convey some environmental information, but these aids are programmed for speech and not for environmental sounds that often have a different frequency range [11–13].

The lack of information about ongoing events makes it difficult for people with DB to comprehend events in their surroundings, creating difficulties in forward planning and control; consequently, they sometimes feel frightened and stressed. People with DB are dependent on others, usually family members or interpreters, for information [10, 14]. The present study with Monitor will deal with environmental perception problems. We will specially focus on “getting to know,” social interaction, safety, and mobility.

In this study Monitor will be evaluated by people with DB using their specific algorithm in a natural environment after a period of training. We decided to evaluate the benefit of Monitor with a group of people of similar age, with a diagnosis leading to DB, who are well educated, able to collaborate and communicate, and who would benefit from a Monitor in their daily lives.

The general purpose of this project is the evaluation of a portable version of Monitor in a field study. This was done by determining the following:

1. the ability to detect and identify ongoing events in their home and traffic environments without Monitor (observation with video recordings);
2. the ability to detect and identify ongoing events in their home and traffic environments with Monitor using an omnidirectional microphone (observation with video recordings);
3. the ability to detect, identify, and recognize the direction of ongoing events in their home and traffic environments with Monitor using a directional microphone (observation with video recordings).

## 2. Methods

We used three field studies with the general aim of evaluating the Monitor.

### 2.1. Participants

People with US I were chosen as participants (Ps) in the study. They are born deaf, have balance problems, have visual adaptation difficulties when they change from bright to dark environments and vice versa due to progressive retinitis pigmentosa (RP), with tunnel vision over time, and have night blindness [15]. Four females between the ages of 44 and 54 years (see Table 1 for anamnestic information) participated in the tests. Written information about the study, procedure, criteria of Ps, and contact information for the test leader was sent to those who met the requirements to determine their interest (Regional Ethics Committee in Uppsala, Sweden, Reg. No. 2006 : 216, revised 2011). Information about Ps' hearings and visions was collected after the test leader received the Ps' consents. The number of Ps is too small for statistical analysis but sufficient for a case study. They are all females (no male with US I reported interest). Three of them have children. They were educated at a high school for people with deafness. The Ps were paid for their participation in the study, which included daily reports of their experiences with Monitor, and performed field tests in their homes. All four Ps communicated by email, SMS, and sign language.

### 2.2. Equipment

#### 2.2.1. Monitor

**Figure 1:**
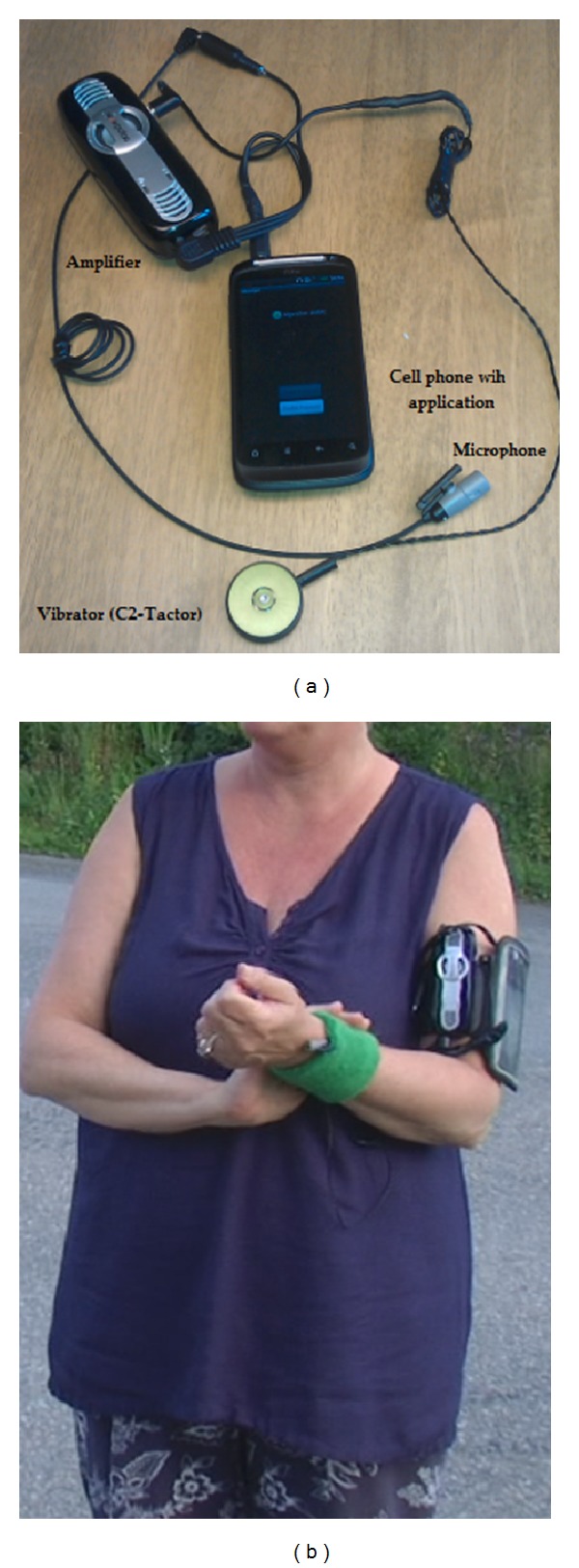
(a) Monitor and its parts, cell phone with the application, microphone, vibrator, and amplifier. (b) The P has the cell phone and the amplifier in the armband, the microphone on the dorsal side of the hand under the sweatband, and the vibrator on the palmar side of the hand under the sweatband. The P uses her right hand to feel the vibrations better.

The vibrotactile aid used in this project, Monitor, consists of a cell phone (HTC based on Android) containing an application, an external microphone, an amplifier (Wowpotas), and a vibrator (see Figure 1). The microphone and the vibrator are connected to the cell phone via the headset (input channel).

#### 2.2.2. Application

The application loaded in the cell phone, Monitor, was programmed with one of the four algorithms (see Table 2) that showed the highest identification for the specific P in a laboratory setting. The four algorithms (AM, AMMC, TR, and TRHA), which had shown optimal results in previous studies, were used to adapt the sound from events to the sensitivity range of the skin [3–6]. The four algorithms, with a short description of each, are shown in Table 2.

The algorithm AM transposed the temporal information in the input signal in the frequency range 0–5500 Hz using a single carrier wave, 250 Hz.

The AMMC algorithm transposed the temporal information in the input signal in the frequency range of 0–5500 Hz, using six carrier waves.

The TR algorithm, transposed the temporal and spectral information in the range of 0–5500 Hz to the range of 0–290 Hz. This algorithm was not used in the field study because it did not show the highest identification score in any P (Ranjbar, in preparation). 

The fourth algorithm was TRHA, which transferred the spectral information to the frequency range of 50–470 Hz by selecting the 10 frequency components with the highest energy every 100 msec in the range of 0–5500 Hz.

#### 2.2.3. Microphone

**Figure 2:**
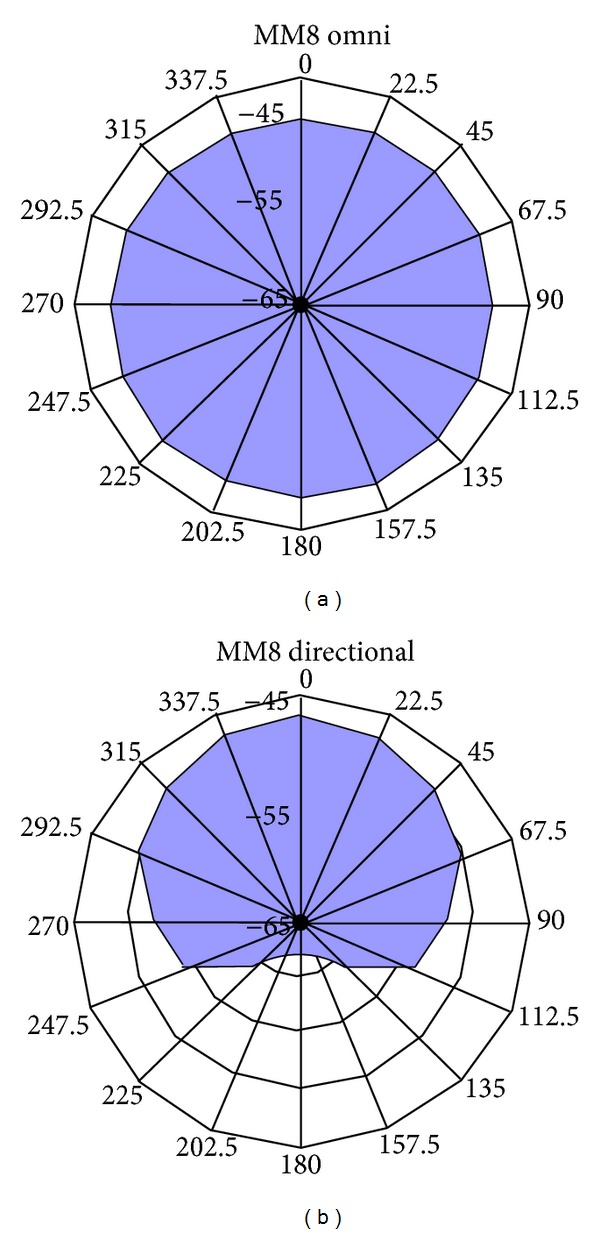
The sensitivity of the Phonac MM8 microphone in two different settings, omnidirectional and directional (adapted with permission from Phonac).

The microphone (Phonac MM8) has two settings, omnidirectional and directional.  Figure 2 shows the sensitivity of the microphone in the two different positions as a function of angle.

The omnidirectional microphone is equally sensitive to sounds from all angles (see Figure 2(a)). In the directional position, it picks up sound from zero degrees azimuth without affecting its level while attenuating the sounds from the sides (see Figure 2(b)).

The microphone was in its omnidirectional setting during training and testing in the first period of the field study and in the directional position in the second period of the field study (see Procedure).

#### 2.2.4. Vibrator

**Figure 3:**
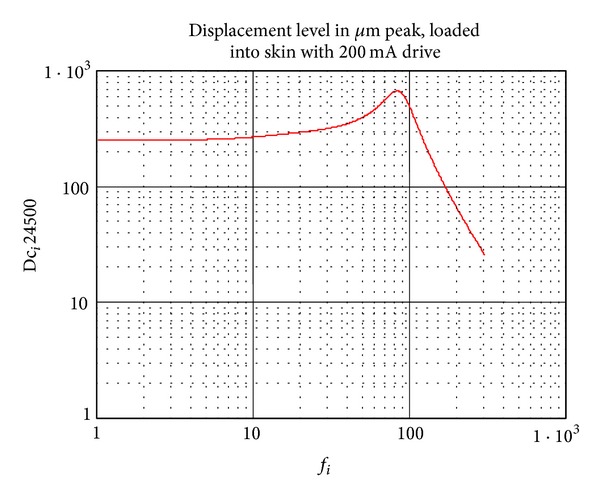
Frequency response of the vibrator C2-tactor. Adapted by permission from http://www.tactors.com/.

The vibrator used by the Ps was a C2-Tactor, and has been used in previous studies [3, 5–7]. The vibrator has a frequency range between 10 and 350 Hz with a peak at 80 Hz (see Figure 3).

#### 2.2.5. Test Stimuli

In the present study, important events producing sounds were selected and presented a different number of times for each P in three different field tests (see further *Procedure*). The events (see Table 3) were selected in previous studies [10] by people with DB and people with normal hearing [4] and in this current study by the Ps as important events to be informed about. The first eight sounds (1–8) in the home environment were the same when testing all Ps in the three different field tests. The remaining sounds (9–15) could be different for the four different Ps or for the same P in the three different field tests. The variations of test stimuli in the home environment were dependent on equipment that the test leaders had access to or the Ps' habits, for example, one is a coffee drinker and uses a coffee maker, while another is a tea drinker and uses an electric kettle.

### 2.3. Procedure

The investigation consisted of three field studies. The first field study included one part, Field test, while the two other field studies included two parts, *training* and field test (documented by video for analysis).

1. Field test without Monitor (noM),
2. field training and test with Monitor with omnidirectional microphone (MO),
3. field training and test with Monitor with directional microphone (MD).

When performing the field tests, the Ps' own technical aids, for example, hearing aid or doorbell detector, were removed.

#### 2.3.1. Field Test without Monitor (noM)

This step included one part: test. The ability of the Ps to perceive their environments was tested at each P's home and traffic environments. Four test leaders (TL1, TL2, TL3, and TL4) were involved in the tests. TL1, TL2, and TL3 initiated different events producing sounds (see Table 3). TL1 also observed and documented the test, and TL4 continuously filmed the test. The events were initiated in random order one or more times using the Ps' own objects. The events were performed in a process, for example, when doing the event “popping popcorn in a microwave,” the door the microwave was opened of the microwave timer was activated and so on. Along with the planned events shown in Table 3, there were also some unplanned events, for example, a person passing.

*Home.* The test was performed in the Ps' own homes, where they were sitting in a relaxed manner with their backs to the test environment to decrease the visual clues of the events. The Ps were encouraged to use all their abilities to detect and identify the ongoing events. The order of the events was determined by a list that was known only to the test leaders. When the Ps detected the event, they signaled their detection by raising their hand and then continued to try to identify the event. The Ps were then also allowed to move and look around and find the sound source and identify the event. During the test, there was a person (an assistant or a relative to the P) with normal vision available who could help the test leaders find the objects that the test leaders needed to produce sound with (e.g., vacuum cleaner). The same person was also allowed to give feedback about the event that the P had detected and identified.

*Traffic.* In the traffic test, the P was walking on a well-known path (e.g., the street between home and her children's school) and using her senses to detect and identify the events (see Table 3). When the P detected the event, she raised up her hand to make clear to the test leaders that she had detected something. After detection, she signaled the identity of the event. In the case of P1, who is blind, her assistant was holding her arm and accompanied her without giving any signals or feedback, necessary to learning [16]. The Ps were allowed to turn and visually search for the detected events in order to identify them.

#### 2.3.2. Field Training and Test with Monitor with Omnidirectional Microphone (MO)

This step included two parts: training and test. The microphone detected sounds from all directions (see Figure 2(a)).

*Training, Daily Use/Report for Five Weeks.* The Ps were instructed to practice as much as they could (waking time) in their residence areas (home, external activities/work, and traffic). The structure of the training was individually different. This made it easier for them to be trained for a longer time instead of the Ps going to the same place and getting trained for 2-3 hours a day. The Ps received written and signed information and instruction about how to handle the equipment and how to send a daily report to the test leader. The number of training days was extended if the P could not be trained, for example, if Monitor was damaged or the P had to take a break. The subject reported daily under the following headings:

day, number of training hours, occasions when Monitor made benefit, occasions when Monitor caused problems, occasions when Monitor made no use, and other comments.

*Test.* After five weeks, the Ps were tested at home and in traffic in the same way, at the same place, and exposed to the same events (random order) as explained in field test *noM. *The difference was that the Ps also used the information from Monitor to detect and identify the events. The events were presented in a random order. Each event was generated as similarly as possible to the previous test when the Ps did not use Monitor. The test was documented with video recordings.

#### 2.3.3. Field Training and Test with Monitor with Directional Microphone (MD)

This step also included two parts: training and test. The microphone was in its directional position so that the detected sounds from behind were attenuated (see Figure 2(b)).

*Training, Daily Use/Report in Five Weeks.* The Ps were trained for another five-week period using Monitor with directional microphone (MD). The P was encouraged to also identify the direction of the events. The Ps continued to send a daily report to the test leader. The conditions were the same as in the earlier five-week training.

*Test.* The Ps were tested at home and in traffic in the same way as field test *noM *andfield test MO. The events were as identical as possible to the previous tests but with a different random order. The test was documented with video recordings.

### 2.4. Analysis Methods

In this case study, each P was analyzed and reported separately. The video recordings from field tests noM, MO, and MD were evaluated. Critical parts were evaluated by two independent observers. A correct detection and identification resulted in one point, and an incorrect response resulted in zero points. A correct response means that Ps signed their detection of the events by raising their hand or they began to identify the event. A correct identification means that the Ps identified the event exactly (not partly) correct.

## 3. Results

### 3.1. Training, Daily Reports with MO/MD

The Ps lived their normal lives and performed their daily activities. In the first training period, they used Monitor (MO) on average of 4–10 hours daily, and in the second period of training, they used Monitor (MD) to average of 3–11 hours daily. The number of training days and average training hours/day for each P for the two training periods are shown in Table 4.

P1, with the longest training period, was the first to begin the training at the end of November. She continued with the training even after completing her five training weeks while waiting for the test leader to organize a test opportunity, which took a long time due to unexpected delays including late equipment delivery and finding a common day convenient for all involved in the test. In the daily reports with Monitor, they described which sounds from events at home and outdoors they had sensed as vibrations (e.g., coffee maker and boiling water). They also reported sounds produced by themselves or others, for example, footsteps, breathing, eating, drinking, own laugh, and laughs from grandchildren. 

The Ps had an increased awareness of sounds they produced thereby helping them control their behavior as an important factor in their social life.

The Ps could detect the people nearby, for example, when one of the Ps sensed speech and discovered that her husband was talking to the dog.

All three Ps with remaining vision had used Monitor when watching TV and discovered such new things as the high volume of music during TV advertising. All four Ps reported music as a new and pleasant experience. 

The information from Monitor could help them have better social interaction and they could act/react, for example, P1 could calm her friend's arguing children. Her reaction surprised the children, who knew that P1 cannot see or hear. P1 also reported that she could sense vibrations when her friend was talking on a cell phone and so P1 kept silent to not disturb her.

Monitor helped them to have better forward planning and save time. They reported better control over household machines, for example, one could turn off boiling water or empty the washing machine when she sensed the vibrations.

Monitor could improve their safety by informing/warning them, for example, one of the Ps could take cover when noting the vibrations of an angry voice. Monitor could also inform them about the presence of cars so they could move away from the car's direction and feel safe.

During the second training period, they had become more curious and explored new sounds, for example, they clapped their hands, they tried to talk (said yes, no, thanks, hello, or goodbye), laughed at sensing their own voices, or they sensed the thunder and rain. P1 reported that the vibrations were stronger when the directional microphone was directed toward the sound source. She could move around with an extended arm with the microphone on and scan the environment to identify the sound source. P3 reported every day that the Monitor was good and she was happy about the information it delivered. Her directional perception had been improved but she could not explain how. Once, she had felt vibrations from a car and for some reason looked at the back of the house where the car was and not in the front, even though cars can drive in the front as well.

Monitor was often of benefit but it also had difficulties, for example, when it was very noisy (when travelling by car or train), the Monitor was vibrating all the time and it was difficult to distinguish between the noise from the train and the voices. A fully charged amplifier could function for a maximum of 13 hours.

The Ps preferred the omnidirectional microphone because they could miss important sounds using the directional microphone. The vibrations of the directional microphone were too weak and difficult to sense when it was not directed toward the sound source and too strong when it was.

They had problems with the cables, for example, one had to use extra tape to keep the Monitor in place and prevent it from dropping. In hot weather, their arms were sweaty. Some Ps thought that the cords were a problem, for example, when cooking food, they were afraid of burning them. Some other Ps felt that the cords were beneficial since they kept all pieces of Monitor in the same place.

All Ps took some break periods due to technical problems or private reasons. No one reported any occasion when the Monitor was of no benefit.

### 3.2. Field Test, noM, MO, and MD

#### 3.2.1. Home Environment

**Figure 4:**
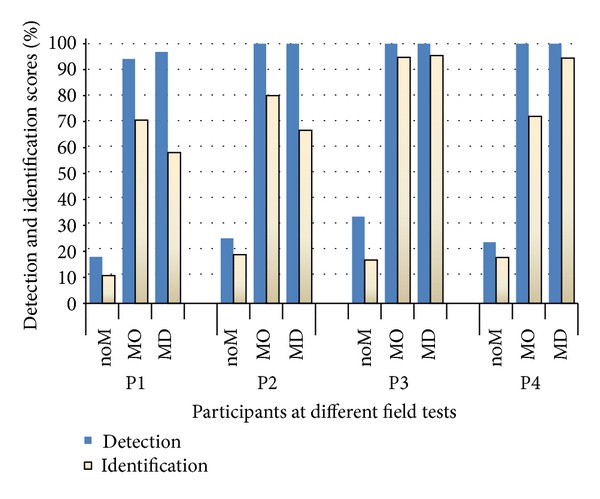
Detection and identification scores of events occurring in the home environment for four participants with Usher's Syndrome type I, when they were tested without Monitor (noM), with Monitor with omnidirectional microphone (MO), and with Monitor with directional microphone (MD).

The detection and identifications results of the three field tests at home for each P are shown in Figure 4.

The numbers of presented test stimuli for each P varied and were as follows:

 field test noM, P1 (28), P2 (16), P3 (18), and P4  (17);
 field test with MO, P1 (17), P2 (20), P3 (19), and P4  (25);
 field test with MD, P1 (31), P2 (15), P3 (22), and P4  (18).

Figure 4 shows that both detection (18%–33%) and identification scores (11%–19%) are lowest when Ps were tested noM. When using MO and MD, the detection scores increased to 100% for P2, P3, and P4 and for P1 to 94% and 97%, respectively.

When using MO, the increments of identification scores were as follows: P1 (60), P2 (81), P3 (78), and P4 (54) percentage units. The corresponding results for test with MD were P1 (47), P2 (48), P3 (78), and P4 (76) percentage units.

*Field Test noM, Details.* The Ps born deaf could all detect the event when other people (test leaders, their assistant, or the interpreter) were passing behind by feeling their air current or smell. The event “door opening and closing” was detected by all of the Ps when the door was closed hard and the Ps felt the cold wind coming in via the door. All the Ps could detect and identify the event “vacuum cleaner,” which was detected when the test leader was cleaning under the chair or touching the chair that the Ps were sitting on (vibratory sense). They could also detect the events “popping popcorn in a microwave oven” and “coffee maker” after the event was finished and they could smell. They did not detect the remaining test events. 

*Field Test MO, Details.* As shown in Figure 4, the detection score of each P was 100% except for P1, whose detection score was 94%. She could not detect the event “water flushing” which occurred in the washroom while she was in the hall (two meters away).

P1 could identify 70% of the events correctly. She identified the event “telephone signaling” as persons laughing which was similar to the signal that was a musical melody. She identified the event “door opening and closing” as “a person sneezing” which is as short and strong as the event “door opening and closing.” 

P2 could correctly identify 80% of the events but confused the event “a person coughing” with the event “dropping keys” which are both short and strong. She could not identify the events “a person talking,” and “dropping keys,”

P3 could identify 94% of the events correctly. She confused the event “door opening and closing” with the event “a person sneezing.”

P4 correctly identified 72% of the events and confused the event “a person coughing” with the events “doorbell,” “water dropping,” or “something strong.” She also confused the event “closing the door” with “a person coughing.” She could not identify the event “a person talking.” 

*Field Test MD, Details.* The detection score of all Ps was 100% except P1 who could not detect “water flushing” in the washroom while she was in the hall two meters away. Ps could identify faster and were more detailed compared to previous tests, for example, P4 could identify the signal produced by the buttons of the timer when conducting the event “popping popcorn in a microwave,” where time was set to 3 minutes, by pushing the buttons. P2 could identify the telephone on the first ring but waited until the second ring to be sure. P1 was often hesitant and changed her mind several times before settling on her final response; therefore, she was tested with more events than the other three Ps. She could identify the direction of a sound source by stretching her arm with the microphone and scanning the area. P3 could identify events without using her vision. When the event “water boiling” was conducted, she responded as “breeze” but changed her response and explained the difference between the two events: “water boiling” starts out faint and becomes stronger over time, but the “breeze” has the same intensity all the time. The only event P4 could not identify was “toilet flushing once” which she explained by the fact that she was expecting that it would flush two times.

#### 3.2.2. Traffic Environment

**Figure 5:**
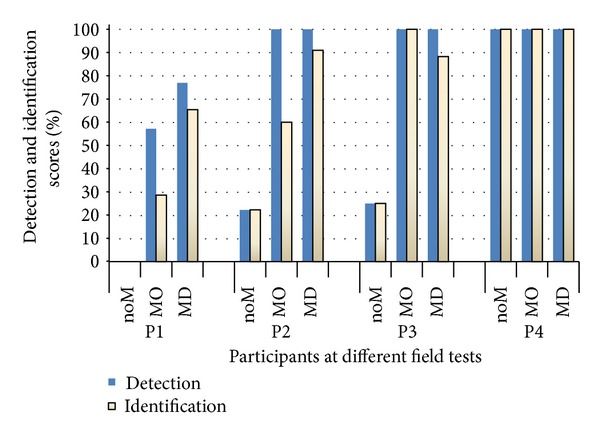
Detection and identification scores of events occurring in a traffic environment for four participants with Usher's Syndrome type I, when they were tested without Monitor (noM), with Monitor with omnidirectional microphone (MO), and with Monitor with directional microphone (MD). In the noM situation the Ps only detected the events coming from behind when the object had passed them.

The detection and identification results of the three field tests in traffic for each P are shown in Figure 5. The numbers of test stimuli for each P varied and were as follows.

Field test noM, P1 (12), P2 (18), P3 (16), and P4 (15); field test MO, P1 (7), P2 (10), P3 (20), and P4 (25); field test MD, P1 (26), P2 (11), P3 (16), and P4 (20).

Figure 5 shows that when testing noM, the detection and identification scores varied from 0% for P1 to 100% for P4, who was able to detect the events when she was five meters or more away, depending on the object—person, car, or bicycle—in front of her. It is to be noted that in the noM situation the Ps only detected the events coming from behind when the object had passed them. They could not act or react other than to be startled. There was no forewarning. Monitor helped to detect and identify the events when approaching from behind and thereby increased their safety. When using MO and MD, the detection scores were 100% for P2, P3, and P4 and 57% for P1. The corresponding identification scores were P1 (29%), P2 (60%), P3 (25%), and P4 (100%).

When using MO, the increment of identification scores was as follows: P1 (29), P2 (38), P3 (75), and P4 (0) percentage units. The corresponding results for test with MD were P1 (65), P2 (31), P3 (63), and P4 (0) percentage units. 

*Field Test noM, Details.* P2, P3, and P4 could detect and identify the person, car, and bicycle coming towards them using their severely restricted visual field (tunnel vision).

**Figure 6:**
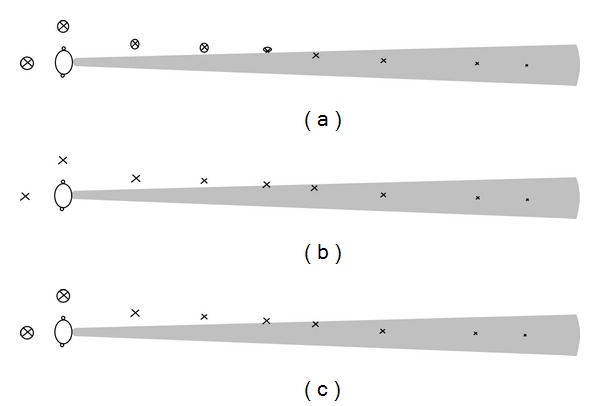
Illustration of a person with tunnel vision in a traffic situation. The shaded area shows the visual field of the P. Objects marked with a cross and ring are not detected while objects marked with a cross are detected. (a) Without Monitor (noM). (b) With Monitor with omnidirectional microphone (MO). (c) With Monitor with directional microphone (MD).

When the person came from behind or was close to their side, the Ps said they were not aware of the event because they could not hear. When the person was close to their side, the Ps said they were not aware because they could not hear and not see due to their tunnel vision. When the person came in front of Ps, he/she was suddenly detected and identified. They are often startled because the person was unexpected. The three situations above are illustrated in Figure 6.

The reaction was the same for the events “car” and “bicycle.” The Ps could detect and identify the car at about 50 meters or more in front and the bicycle at about five meters or more depending on the vehicle's size. 

P3 could not detect the car the first time it was in front of her because its contrast was poor. P1, who has only light perception, could not detect or identify any event. The Ps did not react to any sound such as signal, talk, or running steps.

*Field Test MO.* The detection scores of P2, P3, and P4 were 100%, while P1 scored 57%. P2, P3, and P4 with residual vision could detect events that were several meters outside their visual field. These Ps reported that they could immediately identify the event by the vibrations, but they also turned their eyes towards the sound source to see if they had identified it correctly. They also wanted to show the sound source to the test leaders who were documenting the test on video. 

*Field Test MD.* The Ps could detect all the events (100%) except P1, whose detection score was 77%. P1 often forgot to signal her detection by raising her hand.

The identification scores were as follows: P1 (65%), P2 (90%), P3 (82%), and P4 (100%).

All three Ps (P2, P3, and P4) with residual vision used their vision to identify and localize the events, but in many cases they could recognize the direction of the event without using their visual sense. P2, P3, and P4 could identify the events very quickly.

P2 could identify the events even without using her vision. When she was tested on a sunny day, she was blinded by the sun and had to shade her eyes with her hand, despite sunglasses. Therefore, she could not use her fingers to better sense the vibrations and missed the faint vibrations produced by, for example, the bicycle.

P1 was tested in a path that was noisier than the path of the other three Ps. P1 was sometimes confused and forgot to signal her detection. In some cases, she did not signal but began to identify directly. In those cases when she did not signal her detection, the test leaders were not sure if she did not detect or forgot to signal. She even identified some unplanned events, such as a lawnmower. She could recognize the direction of the lawnmower exactly, which was at least 100 meters from her. She was critical of the directional microphone because its signal becomes low and undetectable when her hand was hanging and the microphone was directed at the ground.

P3 was certain in her identification and could even distinguish between the two different signals produced by the two different bicycles. She could point out the direction of the events with her hand without turning her face to the direction of the sound source.

## 4. Discussion

The focus of the discussion will be on some limitations and implications of the study.

### 4.1. Aspects of Methods

A case study can be designed to have a large variability regarding properties of the Ps: age, cognitive ability, vibratory sensitivity, and training. In the present study, we tried to have a homogenous group.

The participants were selected from the largest diagnostic category of DB, US I. Hearing loss in these individuals is innate, that is, from birth, and thus, they have the same conditions in terms of hearing ability. Participants with other vision and hearing problems would have different conditions and performance. The P can have low sensitivity to vibrations or they can be unmotivated to train, which are important factors affecting the results. It is likely that the Ps were highly motivated since the average time (hours/day) they used the Monitor was high, about 7 hours/day in the first five weeks of training, which increased to 8 hours/day in the second five weeks, since the fully charged amplifier could work a maximum of 13 hours. They appeared very honest and reported with high credibility, for example, P3 reported that she had used the Monitor but forgot to concentrate on the vibrations because she was too busy with household tasks. They sometimes reported that they had a migraine or headache, and therefore, they could not train. In spite of the relative homogeneity of the participating cases, the individual situations varied markedly as well as the use and benefit of the Monitor. It is clear that an individual analysis has to be made regarding the needs and use of this aid as well as most other aids for people with severe sensory impairments [17–19].

The test situations were chosen for having a high validity in the daily life situation of each individual. A complementary study with laboratory tests is in preparation (Ranjbar, in preparation). Few other studies have presented a similar design with video recordings in natural environments and we regard this technique as suitable for analyzing the effect of technical aids in cases of complex impairments [20]. The algorithms used in the study are similar to those used in earlier studies showing equally good results [3–7]. They were simplified to some extent and a further simplification may be needed; it is important to not overload the capacity of the cutaneous senses. On the other hand, systematic use of receptors other than the vibratory (touch and pressure) can increase the transmission and central treatment capacity of the haptic system. The frequency bandwidth of the vibrator we used does not cover the whole range of the vibratory system [21]. A compromise between physical clumsiness and bandwidth is required.

The tool, Monitor, had the same structure for all Ps; the vibrator could be placed where the P is sensitive to vibrations and feels comfortable. As with all other technical equipment, some technical problems occurred when using the Monitor, for example, the cables could be worn or loose, the amplifier had technical failures, the P had unconsciously turned off the volume, and a fully charged amplifier can only function a maximum of 13 hours, which needs to be improved and extended. The cords can be shortened, or a wireless version can be designed in future studies.

Monitor was used and tested in the Ps' residences and in traffic, which was different for each P but made it easier for the P to be trained many hours/day in a real environment without any uncomfortable and awkward changes in their daily life. Also, the variations in the events were greater, and the situations were more natural than if they were trained at the laboratory using the sounds presented via a loudspeaker. This uncontrolled environment could cause some difficulties when testing. For example, when testing P1, the test leaders made coffee using her coffee maker, but the P had not been trained with a coffee machine since she was a tea drinker. On another test occasion, the test leader boiled water in a pot, but the P normally used an electric kettle, which has a different sound. The conditions of the test cases were different for each P, but almost the same for same Ps at different test times (test noM, test MO, and test MD). Even in the traffic environment, the events producing sounds were different on different test occasions, for example, the car was not the same in all tests.

Test sounds were selected by people with DB as representative of the sounds at home and traffic environments in a previous study [4, 10]. They were almost the same for all Ps. The events were easy to perform without disturbing the subjects' privacy too much. The sounds can also vary depending on the source of the sound. For example, a vacuum cleaner or coffee maker can make a sound different depending on the specific device producing the sound. Therefore, tests were done in the P's own residence using the appliances that the P usually uses.

### 4.2. Aspects of Results

#### 4.2.1. Training, Daily Reports

P1, P2, and P4 had been trained for fewer days during the second period field study with MD than the first period, which can be interpreted as reduced interest. The differences can be explained by difficulties when organizing a common test day suited for all people involved when conducting the tests in the first training period. On the other hand, in the second period, they were trained more hours in the same day, indicating increased interest and getting used to handling the Monitor which, according to Ps, became easier with training.

In the beginning, the Ps sensed and reported what they had done in their daily lives; later, they became more curious to explore new sounds like thunder or their own voices. P1 was trained longer than the other three, but still had lower results than them. This can be explained by the fact that she had no useful visual acuity and thereby could not get as much feedback as the others. Her assistants were often people with profound deafness, who could not always inform her about events they had not heard themselves. The volume of P1's Monitor was often too high, which can reduce the dynamic of the vibrator and thereby give a flat pattern to the vibrations. The reduced dynamic could have a more negative effect, especially in noisy environments such as in traffic where P1 was tested. An effective and standard training program similar to those used for cochlear implant and other aids should be designed and tested in future studies [17].

#### 4.2.2. Field Tests

Both detection and identification scores were high. The detection scores of Ps with residual vision were 100% both at home and in traffic with Monitor with MO/MD. The Ps had also high identification scores (>67%) with Monitor. They confused events with similar signal patterns. The results in traffic were affected by difficult weather conditions, for example, it was windy and the vibrator vibrated all the time, or it was −15 degrees, and therefore, the P could not use her fingers to feel the vibrations better. The Ps also had balance problems and had to focus on walking. Monitor was evaluated in a realistic environment and should become more robust for different conditions. An algorithm with noise reduction and automatic volume adaptation should be developed.

The directional microphone delivered directional information but could also miss some important sounds. A new version with a combined directional and omnidirectional microphone should be designed.

Monitor was able to inform the Ps about ongoing events producing sounds, resulting in increased forward planning, improved feeling of safety, increased social interaction, and increased mobility. Monitor enhanced the benefit they got from their remaining vision, especially in traffic. As a disadvantage, they mentioned that Monitor cannot detect events that do not produce sounds, which is a problem. The residential area of DB people is very often quiet, for example, people nearby do not talk loudly because the Ps cannot hear. The environment should be adapted as for people with normal hearing, for example, the people nearby should use more of their voices to get the deafblind person's attention instead of just tapping them.

The Ps had improved directional recognition. They could even detect events that were behind them and out of their field of vision, especially in traffic. They could frequently and more easily use their vision to recognize the direction of events or by scanning the environment to feel where the sound was stronger, indicating a directional correction toward the sound source. Using the directional microphone, they could also recognize the direction of simultaneous sounds from different sources. In some cases, they could miss important things because the microphone was not directed at them. An alternative construction of the directional microphone is of interest in future studies.

Monitor improved their social interaction. By sensing talk from other people, they could keep quiet until it was their turn to talk, or simply not bother them. They could control their own body sounds, for example, when eating, talking loudly, or signing to a deaf person.

Monitor improved their mobility by informing them about approaching cars, motorcycles, running persons and so on so they could move out of the way. If they dropped something they could detect and look for it and not stumble on it.

They had reduced stress because they could get information about events in advance, resulting in improved forward planning.

They thought that using Monitor was interesting, and they curiously explored new sounds with high motivation even after their five-week training, while waiting to be tested.

## 5. Conclusions and Further Developments

Monitor, the vibrotactile aid for environmental perception, could improve the ability of people with DB to detect, identify, and recognize the direction of ongoing events producing sounds. The results showed that Monitor could improve their mobility, social interaction, forward planning, and feeling of safety and decrease their feelings of stress.

A further development should be focused on the following.

A new version with a combined directional and omnidirectional microphone should be designed to deliver both the identity of the sound without attenuating and the directional information.

Training programs comparable to cochlear implant training programs should be developed to increase the effectiveness of environmental sound identification.

The design of Monitor should be improved by making it wireless.

## Figures and Tables

**Table 1:** Anamnestic information of the four female participants with Usher's Syndrome I (P1, P2, P3, and P4).

Participant	P1	P2	P3	P4
Age	50	44	51	54
Visual Acuity (right eye)	Light perception (yr 2009)	0.1 cc (yr 2011)	0.3 cc (yr 2013)	0.16 cc (yr 2011)
Visual Acuity (left eye)	Light perception (yr 2009)	0.09 cc (yr 2011)	0.4 cc (yr 2012)	0.16 cc (yr 2011)
Visual field, right eye (Goldman, V/4 obj.)	<2°(yr 2009)	5° (yr 2011)	<10° (yr 2012)	10° (yr 2011)
Visual field, left eye (Goldman, V/4 obj.)	<5° (yr 2009)	5° (yr 2011)	<10° (yr 2012)	10° (yr 2011)
Age of subjective notified visual impairment	About eight	Teens	Teens	Teens
Hearing	Born deaf	Born deaf	Born deaf (used hearing aid)	Born deaf
Ways of communication	Tactile sign language, E-mail, and SMS and, braille	Visual and Tactile sign language, E-mail, SMS, reading, and writing	Visual sign language, E-mail SMS, reading, and writing,	Visual sign language, E-mail SMS, reading, and writing,

**Table 2:** The four algorithms used to process the sounds in the laboratory study.

Abbreviation	Description
AM	Amplitude modulation of a 250 Hz carrier wave.
AMMC	Amplitude modulation with multiple channels.
TR	Transferring data in the range of 0–5500 Hz to the range of 0–290 Hz.
TRHA	Transposing the 10 frequency components with highest amplitude in the range of 0–5500 Hz to the range of 52–470 Hz.

**Table 3:** Sounds from events used in the tests in home and traffic environments.

Number	Sounds from events in home environment
(1)	Doorbell
(2)	Water flushing
(3)	Telephone signaling
(4)	Toilet flushing
(5)	Door opening and closing
(6)	Popping popcorn in a microwave oven
(7)	Vacuum cleaner
(8)	A person talking
(9)	Coffee maker
(10)	Talk and music from TV
(11)	Dropping keys
(12)	Footsteps
(13)	Heavy traffic from window
(14)	Water boiling
(15)	A person coughing

Number	Sounds from events in traffic environment

(1)	Bicycle passing from behind with/without signaling
(2)	Bicycle coming towards P with/without signaling
(3)	Car passing from behind with/without signaling
(4)	Car coming towards P with/without signaling
(5)	A person running from behind to front
(6)	A talking person walking from behind to front

**Table 4:** The number of training days and average training hours/day (digits in the parenthesis) for each P at two-training period.

	P1	P2	P3	P4
Five weeks training with MO	109 (9.6)	33 (4.0)	25 (5.6)	57 (8.9)
Five weeks training with MD	33 (11.1)	21 (7.0)	32 (2.7)	35 (10.7)
